# Book Reviews

**Published:** 1893-03

**Authors:** 


What to Do in Case of Accident. By Merrill B. Rickets, Ph. B., M. D., Member of the Ninth International Medical Congress (1887); American Medical Association; Ohio State Medical Society; Fellow of the New York State Medical Association, Mississippi Valley Medical Association, National Association of Railway Surgeons, Cincinnati Academy of Medicine, Cincinnati Medical Society, Southwestern Ohio Medical Society, Cincinnati Natural History Society; Visiting Dermatologist, German Protestant Hospital; Physician for Skin Diseases and Plastic Surgery to Christ Hospital, and Consulting Physician for Skin Diseases to Ohio State Hospital and Woman's Medical College; Chief Surgeon for C. P. & V. R. R. and A. E. & L. Ins. Co. Cincinnati: Printed by James Barcley, 124 Canal street. 1892.

In this little volume of sixty-one pages Dr. Rickets has concentrated an immense amount of useful information on the subject on which it treats. There is hardly a single form of danger from sudden attack of illness, whether from poison, traumatism, or zymotic disease, that is not met by sensible, scientific, and simple remedial agents, which may be applied or administered by any cool and intelligent person till the physician, who, of course, should be immediately summoned, can arrive.

We all know that there are times when even a little knowledge and common sense may save life if immediate resort to the proper means be taken, such as, for instance, in cases of drowning, bleeding, croup, poisoning, and a host of other conditions where prompt aid is necessary. Careful directions, with very easily understood pictorial illustrations, are given in cases of accidents requiring surgical treatment, showing how and where to apply bandages and tourniquets. Diet and food receive proper attention, and the list of antidotes in cases of poisoning ought to be studied and committed to memory by every one having a family in charge. Much useful information not directly connected with the general subject is furnished. The book may, indeed, be called a *multum in parvo* of special benefit to families in places remote from medical aid.

A Hand-Book of Hygiene and Sanitary Science. By George Wilson, M. A., M. D., F. R. S., Edin., D. P. H., Camb.; Fellow of the Chemical Society; Fellow of the Sanitary Institution of Great Britain; Medical Officer of Health for the Mid-Warwickshire Sanitary District; formerly Medical Officer, H. M. female prison, Woking, and H. M. convict prison, Portsmouth. Seventh edition. Philadelphia: P. Blakiston, Son & Co., 1012 Walnut street. 1892. Price, $3.25.

This author has long been before the public as an accepted teacher on the subjects treated upon in this book, hence his seventh edition needs only to be noticed to insure its purchase. It has been out of print for more than a year, but has now come to us with a revision and completeness that brings it up to the present date in the important subjects of which it treats. Much of the work has been rewritten and the book has been enlarged by more than 200 pages. Besides, a number of new wood-cuts have been added, which serve to further perfect the work. There is such a marked tendency in the present age among all physicians to study, under competent masters, the science of prevention as well as cure, that a vast increase in the sale of sanitary and hygienic literature has taken place within a short time. Many of the treatises on these subjects are large and exhaustive, but we know of none more practical for the every-day physician than Wilson's Hand-Book. We hope it will be purchased and studied, especially by health officers of towns throughout the State. These duties are frequently performed in a perfunctory manner by busy doctors, but they should change front and make their services more valuable to the people. Let them purchase this book, and be stimulated thereby to better work.

---

ADDRESSES AND ESSAYS. By G. FRANK LYDSTON, M. D., Professor of the Surgical Diseases of the Genito-Urinary Organs and Syphilology, in the Chicago College of Physicians and Surgeons; Surgeon-in-Chief of the Genito-Urinary and Venereal Department of the West Side Dispensary, Chicago; Fellow of the Chicago Academy of Medicine, and of the Southern Surgical and Gynecological Association; Lecturer on Criminal Anthropology in the Union Law School; Honorary Member of the Texas State Medical Association. Second edition, revised and enlarged. Renz & Henry, Louisville, Ky. 1892.

Under this title this interesting and accomplished author has grouped a number of chapters, for the most part relating to Diseases of the Genito-Urinary System in the Male and Female. The fact that a second edition has so soon been called for, speaks in decided language as to the value of this book. The profession is already familiar with these essays and lectures, hence we need only call attention to the fact that the second edition has been published and is now offered to the profession. We presume it will be promptly exhausted, hence, those who desire to obtain the book must make early application to the trade therefor.

---

KIRKE'S HAND-BOOK OF PHYSIOLOGY. By W. MORRANT BAKER, F. R.C. S., Late Surgeon to, and Lecturer on, Physiology at St. Bartholomew's Hospital, etc., and Vincent Dormer Harris, M. D., London, Examiner of Physiology at the Conjoint Board of the Royal College of Physicians and Surgeons, and in the University of Durham; Demonstrator of Physiology at St. Bartholomew's Hospital; Physician to the Victoria Park Hospital for the Diseases of the Chest. Thirteenth edition. With upwards of 500 illustrations, including some colored plates. Philadelphia: P. Blakiston, Son & Co., 1012 Walnut street. 1892.

This standard work now appears in its thirteenth edition, and still retains its hold upon the medical student as one of the best of its kind. Since the last edition, the book has undergone a thorough revision, and its scope has been somewhat enlarged, but its general value to the student has not been impaired, but rather increased. The object of this book is to study physiology only as an introduction to medicine, as announced in the preface, and we know of no work that more nearly fulfils its mission in this respect. It is beautifully illustrated and printed on handsome paper, and though the work contains 884 pages it is not cumbersome, but can easily be carried back and forth between the laboratory and study.

